# Activation of a T-box-Otx2-Gsc gene network independent of TBP and TBP-related factors

**DOI:** 10.1242/dev.127936

**Published:** 2016-04-15

**Authors:** Emese Gazdag, Ulrike G. Jacobi, Ila van Kruijsbergen, Daniel L. Weeks, Gert Jan C. Veenstra

**Affiliations:** 1 Department of Molecular Developmental Biology, Radboud Institute for Molecular Life Sciences, Radboud University, 6500 HBNijmegen, The Netherlands; 2 Department of Biochemistry, University of Iowa, Iowa City, IA 52242, USA

**Keywords:** TATA-binding protein, Gene regulation, *Xenopus*, Organizer, Mesoderm

## Abstract

Embryonic development relies on activating and repressing regulatory influences that are faithfully integrated at the core promoter of individual genes. In vertebrates, the basal machinery recognizing the core promoter includes TATA-binding protein (TBP) and two TBP-related factors. In *Xenopus* embryos, the three TBP family factors are all essential for development and are required for expression of distinct subsets of genes. Here, we report on a non-canonical TBP family-insensitive (TFI) mechanism of transcription initiation that involves mesoderm and organizer gene expression. Using TBP family single- and triple-knockdown experiments, α-amanitin treatment, transcriptome profiling and chromatin immunoprecipitation, we found that TFI gene expression cannot be explained by functional redundancy, is supported by active transcription and shows normal recruitment of the initiating form of RNA polymerase II to the promoter. Strikingly, recruitment of Gcn5 (also known as Kat2a), a co-activator that has been implicated in transcription initiation, to TFI gene promoters is increased upon depletion of TBP family factors. TFI genes are part of a densely connected TBP family-insensitive T-box-Otx2-Gsc interaction network. The results indicate that this network of genes bound by Vegt, Eomes, Otx2 and Gsc utilizes a novel, flexible and non-canonical mechanism of transcription that does not require TBP or TBP-related factors.

## Abstract (precis)

**Highlighted article:** A network of embryonic genes, many of which are expressed in the mesoderm and the organiser, can initiate transcription through a non-canonical mechanism.

## INTRODUCTION

The gene expression patterns underlying embryonic development are orchestrated by highly complex gene regulatory networks. Compared with single-cell eukaryotes, metazoans have expanded repertoires of protein-coding genes involved in transcriptional regulation to support these networks (Tupler et al., 2001), but how this diversified transcription machinery is adapted to developmental gene expression is not yet fully understood.

The step-wise mechanism of transcription initiation is known to start with binding of TFIID, a complex of TATA-binding protein (TBP) and TBP-associated factors (TAFs). In this canonical mechanism of transcription initiation TBP binding represents a rate-limiting step before the recruitment of RNA polymerase II (RNAPII) to the core promoter of protein-coding genes (Kim and Iyer, 2004; Klein and Struhl, 1994; Wu and Chiang, 2001). However, the role of TBP is not universal and additional TBP-related factors have been discovered in metazoan genomes (reviewed in Akhtar and Veenstra, 2011; Müller et al., 2010). TBP-related factor (TRF1) was discovered in *Drosophila* and has only been found in insects (Crowley et al., 1993; Hansen et al., 1997). In vertebrates, the TBP family comprises TBP (present in archaea and all eukaryotes), TBP-like factor (TLF; also known as TBPL1/TRF2/TLP; present in all metazoans) and TATA-binding protein 2 (TBP2; also known as TBPL2/TRF3; unique to vertebrates) (Akhtar and Veenstra, 2011). TLF is essential for embryogenesis in *Caenorhabditis* *elegans*, *Drosophila*, zebrafish and *Xenopus* and for spermatogenesis in mouse (Dantonel et al., 2000; Hart et al., 2007; Kaltenbach et al., 2000; Kopytova et al., 2006; Martianov et al., 2002a; Müller et al., 2001; Veenstra et al., 2000; Zhang et al., 2001). TBP2, which is most closely related to TBP, is required for embryonic development in zebrafish and *Xenopus*, and for oogenesis in mouse (Bártfai et al., 2004; Gazdag et al., 2009; Hart et al., 2007; Jallow et al., 2004). Core initiation factor switching can be mediated by changes in initiation factor expression (Akhtar and Veenstra, 2009; Jallow et al., 2004). TBP2 replaces TBP as the major initiation factor in oocytes because of an abundance of TBP2 and a lack of TBP. TBP2 is degraded during meiotic maturation (Akhtar and Veenstra, 2009). TBP is virtually absent in oocytes but is translated from maternal stores of mRNA just before the mid-blastula transition when embryonic transcription starts (Gazdag et al., 2007; Veenstra et al., 1999). TBP, TLF and TBP2 regulate different subsets of transcripts during gastrulation and these transcripts functionally link TLF and TBP2 to the metazoan and vertebrate developmental programs (Jacobi et al., 2007).

It is not known however, whether the diversity brought about by TBP family members encompasses all existing initiation mechanisms, or if additional mechanisms exist that are independent of TBP and TBP-related factors. In mouse zygotes, abundant transcription occurs in the absence of TBP without any indication of rescue by the other two TBP-related factors (Martianov et al., 2002b), suggesting that additional mechanisms do exist. Moreover, a human TAF-containing TBP-free complex (TFTC) has been reported to support transcription initiation *in vitro* (Wieczorek et al., 1998). TFTC is similar to the yeast Spt-Ada-Gcn5-acetyltransferase complex (SAGA) and has a conserved subunit composition across species (Spedale et al., 2012; Wang and Dent, 2014; Wieczorek et al., 1998). This Gcn5-containing complex interacts with TBP but is not stably associated with it (Larschan and Winston, 2001). The histone fold-containing TAF and TAF-like subunits of the complex form a TFIID-like structure (Han et al., 2014). Moreover, like TFIID, SAGA is a reader of the promoter-associated H3K4me3 histone mark (Vermeulen et al., 2010) and is a cofactor of RNAPII-dependent transcription (Bonnet et al., 2014; Nagy et al., 2010).

Here, we investigate to what extent TBP family-independent initiation mechanisms are involved in embryonic gene regulation. We address this question in *Xenopus* embryos by ablation of mRNA encoding TBP, TLF and TBP2 from embryos. Strikingly, our analyses uncover a network of genes that are robustly induced during gastrulation and which recruit RNAPII to the promoter under TBP family triple-knockdown conditions. These data provide new insight into the diversity of transcription initiation and identify a robustly activated embryonic gene network that is supported by a non-canonical mechanism independent of TBP, TLF or TBP2.

## RESULTS

### TBP family-insensitive gene transcription in early *Xenopus* embryos

**Fig. 1.**
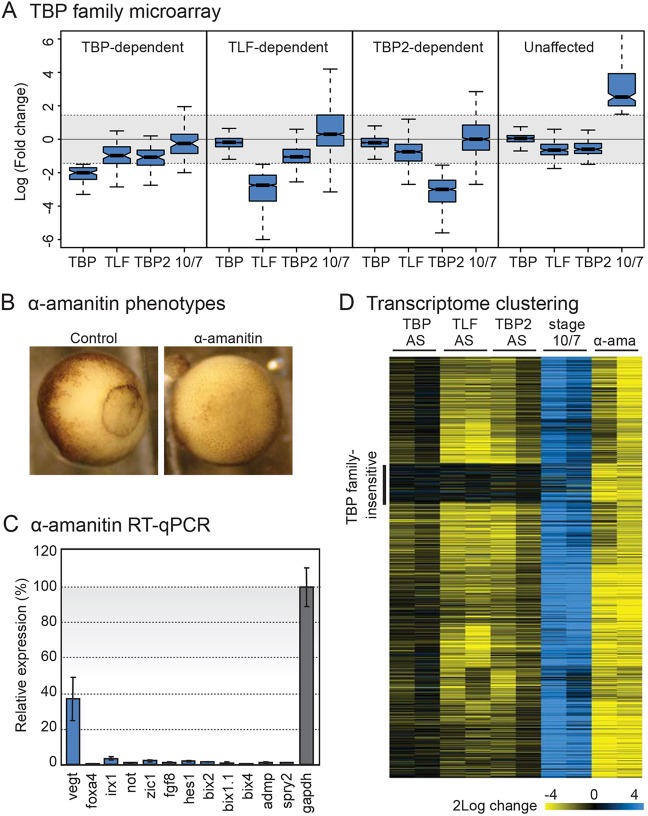
**Analyses of *tbp*, *tlf* and *tbp2* mRNA ablation, embryonic gene activation and α-amanitin treatment reveal TBP family-insensitive gene transcripts.** (A) Box plots of the fold change of groups of transcripts upon TBP, TLF or TBP2 knockdown as measured by microarray. Expression ratio of stages 10.5 (early gastrula) and 7 (blastula) is also plotted (10/7). TBP-, TLF- and TBP2-dependent transcripts (first three panels) were identified using a consistent statistical change call between replicates for the initiation factor that was ablated, in addition to a fold change (log_2_ ratio<−1.5) in expression. The unaffected group of transcripts (fourth panel) was identified by selecting for developmentally upregulated transcripts (between stage 7 and 10.5) with no significant change upon injection of TBP family antisense oligos. Boxplots are standard box plots generated in R, showing the interquartile range (IQR) around the median; the whiskers extend from the minimum value to the maximum value unless the distance to the first and third quartiles is more than 1.5 times the IQR. The fold change cut off range is shaded gray in each panel. (B) Phenotype of control and α-amanitin-treated embryos at stage 10.5. (C) Effect of α-amanitin treatment on transcript levels of ‘unaffected’ TFI genes, demonstrating that these transcripts are newly made in the embryo, with the exception of *gapdh* (maternally provided control) and *vegt* which is also partly maternally provided. (D) K-means clustering of transcript expression ratios [antisense oligo-treated relative to control; stage 10.5 relative to stage 7; α-amanitin (α-ama) relative to control] of developmentally active transcripts (defined as transcripts that are either developmentally upregulated or affected by α-amanitin at stage 10.5). Each column represents one of two replicate experiments. A cluster of genes insensitive for TBP, TLF, or TBP2 depletion is marked on the left.

TBP, TLF and TBP2 are all essential for gastrulation and for transcription of partially overlapping subsets of genes in *Xenopus laevis* embryos (Jacobi et al., 2007; Jallow et al., 2004; Veenstra et al., 2000). We asked whether all actively transcribed genes require TBP or one of the TBP family members. In the transcriptome of TBP, TLF and TBP2 knockdown embryos (Jacobi et al., 2007), specific subsets of transcripts can be identified requiring one of these factors in early development (Fig. 1A). Early embryos are loaded with maternal transcripts, many of which are gradually replenished after zygotic genome activation at the mid-blastula stage (stage 8.5). Yet, many of these maternal transcripts are sustained until the end of gastrulation without new transcription. For an analysis of initiation factor requirements, it is therefore important to consider only transcripts that are actively transcribed. Developmentally induced transcripts were identified using statistical change calls (Wilcoxon *P*<0.05, consistent between replicates) of early blastula (stage 7) and early gastrula (stage 10.5) embryos, with additional filtering for more than 2.8-fold (log_2_ 1.5) change in expression between these stages. Strikingly, among these developmentally activated transcripts, a total of 180 were not affected by ablation of TBP, TLF or TBP2 (Fig. 1A, Table S1), raising the possibility that some transcripts do not require these factors for the rate-limiting step of transcription initiation.

To test whether the transcripts involved were actively transcribed, α-amanitin was injected into fertilized eggs to inhibit RNAPII. Embryonic transcription is required for the onset of gastrulation (Newport and Kirschner, 1982; Sible et al., 1997) and its inhibition by α-amanitin interferes with the appearance of the blastopore (Fig. 1B). mRNA expression of the set of genes that were unaffected upon knockdown of TBP, TLF or TBP2 (Fig. 1A) was analyzed by RT-qPCR in the presence of α-amanitin. Most of these transcripts were reduced to less than 5% of control levels (Fig. 1C), indicating that these mRNAs are indeed actively transcribed in the embryo. The T-box transcription factor gene *vegt*, which is expressed both maternally and zygotically (Fukuda et al., 2010), was reduced but to a lesser extent than the other genes. The maternally expressed *gapdh* gene was used as a control and its levels were unaffected by α-amanitin. To extend the analysis to all transcripts that depend on new transcription rather than maternal stores, microarray analysis of α-amanitin-treated and control embryos was performed in duplicate. K-means clustering was performed on the relative expression ratios of transcripts (Fig. 1D). Almost all transcripts that were lost in α-amanitin-injected embryos were also strongly induced between early blastula and gastrula stages; the vast majority of these genes were transcriptionally impaired under TBP, TLF or TBP2 knockdown conditions. The exception was a conspicuous cluster of transcripts that, although generated by active transcription and strongly induced during development, were virtually unaffected by knockdown of TBP, TLF or TBP2. These results indicate the presence of a relatively small subset of genes for which normal levels of TBP, TLF or TBP2 are not rate-limiting for transcription. For further analysis, a set of 205 transcripts was selected that (1) were not affected by knockdown of TBP, TLF or TBP2, and (2) were either induced during development (Fig. 1A) or were sensitive to α-amanitin (Fig. 1D). This set includes the 180 developmentally induced transcripts described above, but also includes transcripts that were not strongly induced but required transcription to be maintained in the early gastrula embryo. We will refer to these transcripts as TBP family-insensitive (TFI, Table S1).

Together, these results indicate that a subset of actively transcribed developmental genes do not require normal levels of TBP, TLF or TBP2 for transcription. However, these experiments were performed with individual knockdowns of each of the TBP family members, raising the question whether TBP family members could act redundantly.

### RNAPII is recruited to TFI gene promoters in TBP family triple-knockdown embryos

**Fig. 2.**
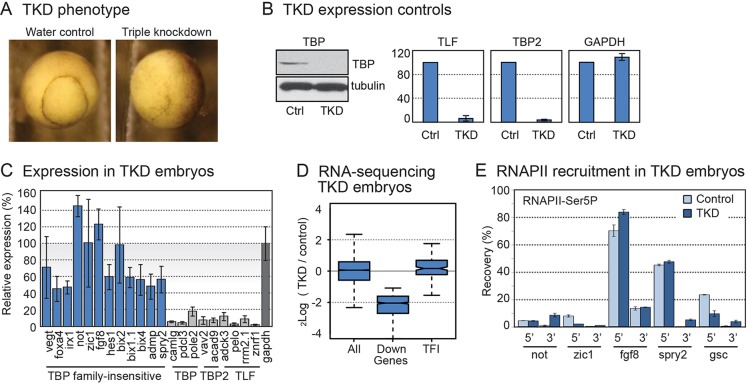
**Analysis of TBP family triple knockdown (ablation of *tbp*, *tlf* and *tbp2* mRNA).** (A) Morphology of TKD embryos at stage 10.5, relative to water-injected control (left panel). (B) Control experiments verified efficient TBP protein depletion (western blot, left panel) and knockdown of *tlf* and *tbp2* transcripts by RT-qPCR in TKD embryos relative to control (Ctrl). Tubulin (western) and *gapdh* serve as controls. (C) Expression levels of TFI gene transcripts (left, in blue) and transcripts that require TBP, TBP2 or TLF (light gray) in TKD embryos relative to control embryos, as determined by RT-qPCR. *gapdh* transcript levels (dark gray) served as control. (D) Box plots showing fold change (log_2_) of transcript levels of all expressed genes, decreased genes (DEseq FDR 0.1, see Materials and Methods) and TFI genes, as determined by RNA sequencing of ribosomal RNA-depleted samples of TKD embryos versus control embryos. (E) Recruitment of initiating RNAPII-Ser5 determined by ChIP-qPCR. 5′ and 3′ ends of TFI genes were analyzed. Comparison of signals obtained from control (light blue) and TKD embryos (dark blue) indicates significant recruitment of RNAPII-Ser5 to TFI genes under TKD conditions.

To test possible redundant functions of TBP, TBP2 and TLF in the transcription of TFI genes, we depleted all three family members by microinjecting a combination of *tbp*, *tbp2* and *tlf* antisense oligonucleotides. We used DMED-modified oligonucleotides that lead to RNaseH-mediated degradation of the targeted mRNA (Dagle and Weeks, 2001). The specificity of the TBP, TBP2 and TLF knockdown reagents has been established with control oligonucleotides and rescue experiments, and the single-knockdown phenotypes have been described previously (Jacobi et al., 2007; Jallow et al., 2004; Veenstra et al., 2000). Morphologically, triple-knockdown (TKD) embryos were undistinguishable from controls until stage 9, but from stage 10 they arrested and did not enter gastrulation. Some pigmentation appeared at the site of the blastopore; however, there were no signs of cell involution (Fig. 2A). We verified TBP depletion at the protein level by western blotting (Fig. 2B, left panel) and analyzed depletion of *tbp2* and *tlf* mRNA by RT-qPCR (Fig. 2B, right panel). Next, we continued to examine active gene expression in TKD embryos. TFI genes that we tested for RNAPII requirement in α-amanitin-injected embryos appeared to be actively transcribed under TKD conditions, although some were expressed at slightly lower levels in these arrested embryos compared with controls (Fig. 2C). By contrast, transcript levels of genes that were originally identified to be dependent on TBP, TLF or TBP2 were severely reduced. To examine the genome-wide potential for redundancy, we performed RNA sequencing in duplicate samples of TKD and control embryos, mapped the results to the recently released *X. laevis* genome and identified which genes were affected by loss of TBP family members (see Materials and Methods and Table S2). The full set of TFI genes was hardly affected by TBP family TKD (Fig. 2D), with transcript levels even slightly increased (Wilcoxon signed rank *P*-value 0.0022). At a false discovery rate of 10%, only four TFI genes were decreased (*srsf2*, *hnf1b.L*, *hnf1b.S* and *slc7a5*). The finding that TFI genes are robustly transcribed under TKD conditions suggests that none of the TBP-related factors are required for the rate-limiting step of transcription of these genes and that functional redundancy of TBP family of initiation factors does not play a major role in this process.

A prominent role has been identified for promoter-proximal pausing of RNAPII in eukaryotic gene regulation (Adelman and Lis, 2012). This raised the possibility that enhanced elongation compensates for reduced transcription initiation, rescuing the expression of TFI genes under knockdown conditions. We tested this hypothesis by examining the recruitment of initiating RNAPII to promoters by chromatin immunoprecipitation (ChIP) of the Ser5-phosphorylated form of RNAPII (Fig. 2E). In TKD embryos, RNAPII phosphorylated at Ser5 is unaffected at robustly transcribed promoters such as *fgf8* and *spry2* but moderately decreased at genes such as *zic1* and *gsc*. Assessing RNAPII recruitment using an antibody recognizing the unphosphorylated CTD domain of RNAPII did not uncover altered RNAPII recruitment to TFI genes in TKD embryos (Fig. S1A). Concordantly, analysis of RNAPII pausing at TFI orthologues using *X. tropicalis* RNAPII ChIP-seq data (van Heeringen et al., 2014) did not reveal significant pausing of TFI genes relative to other expressed genes (Fig. S1B). Together, the results did not reveal a role for post-initiation regulation in TFI transcription but rather highlighted the fact that the initiating form of RNAPII can be robustly recruited at TFI genes under TKD conditions.

### Role of TFI genes in mesoderm, the organizer and other developmental processes

**Fig. 3.**
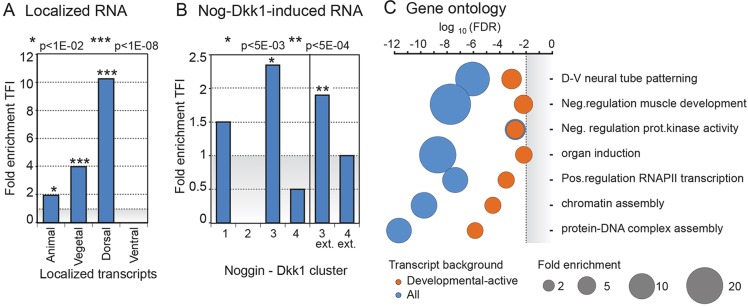
**Characterization of TFI gene transcript localization and function.** (A) Over-representation (fold enrichment) of TFI gene transcripts among localized expression in the animal, vegetal, dorsal and ventral parts of the embryo. TFI transcripts are not over-represented among ventrally expressed transcripts. Hypergeometric *P*-values are indicated. (B) Over-representation of TFI gene transcripts within four clusters of genes identified after ectopic induction of the organizer by Noggin (Nog) and Dickkopf-1 (Dkk1). Clusters 3 and 3-extended represent organizer genes (Hufton et al., 2006). TFI gene transcripts are moderately but significantly over-represented in these clusters. (C) Gene ontology (GO) term analysis of TFI genes relative to GO terms in all genes (blue) or in developmentally active genes (orange, defined as in Fig. 1D). The GO fold enrichments are reflected in the sizes of the circles (see scale in gray). Significance (FDR; top) and relevant GO terms (right) are indicated.

We noticed that many TFI gene transcripts have developmental functions in both mesendoderm specification and the Spemann-Mangold organizer, including well-known genes such as *goosecoid* (*gsc*), *otx2*, *foxd3*, *notochord homeobox* (*not*), *zic1*, *noggin* (*nog*), *spry2*, *admp*, *eomesodermin* (*eomes*) and *vegt*. Therefore, we investigated the functional characteristics of the TFI genes. We assessed the spatial localization of TFI gene transcripts using available transcriptome datasets of dissected embryonic tissue of early gastrula (stage 10) embryos, including dorsal blastopore lip (dorsal), ventral marginal zone (ventral), animal cap (animal) and the central part of the yolk plug (vegetal) (Tanegashima et al., 2009; Zhao et al., 2008). Among 15,491 transcripts, 466 were expressed more than 2.8-fold (log_2_ 1.5) higher at the animal pole compared with the vegetal pole. Of these transcripts 12 were TFI (1.9 times more than expected by random chance, hypergeometric *P*-value 9.3×10^−3^, Fig. 3A). Twenty-five TFI gene transcripts were more abundant at the vegetal pole than at the animal pole (fourfold enriched, *P*-value 8.2×10^−10^), and at the dorsal blastopore lip eight transcripts were enriched relative to the ventral marginal zone (10.2-fold, *P*-value 7.4×10^−8^). By contrast, no TFI gene transcripts overlapped with the 43 transcripts that were more highly expressed at the ventral marginal zone compared with the dorsal blastopore lip (Fig. 3A). This over-representation of TFI gene transcripts among transcripts with dorsal-vegetal gene expression is consistent with roles in mesoderm and the organizer. To determine whether TFI genes were enriched for organizer genes, we investigated the overlap with Noggin-Dickkopf (*nog-dkk1*)-induced transcripts, which represent transcripts induced by an ectopic organizer (Hufton et al., 2006). Four clusters of transcripts have been identified, of which clusters 3 and 4 correspond to organizer and ventral-posteriorizing transcripts, respectively. TFI gene transcripts were enriched in the organizer cluster 3 (2.3-fold compared with all developmentally upregulated transcripts; *P*-value, 2×10^−3^). Also, an extended organizer cluster (Hufton et al., 2006) was enriched with a total of 18 TFI gene transcripts (Fig. 3B). To characterize the biological processes to which TFI gene transcripts contribute, gene ontology (GO) analysis was performed using all genes and developmentally upregulated genes as background gene lists (Fig. 3C, Table S3). TFI genes were enriched in functional categories linked with dorso-ventral neural tube patterning, negative regulation of muscle development, induction of organ formation, regulation of transcription, chromatin assembly and DNA-protein complex assembly. This illustrates that, apart from mesoderm and organizer gene expression, TFI genes also contribute to other developmental processes.

### Role of Gcn5 in gastrulation and embryonic transcription

**Fig. 4.**
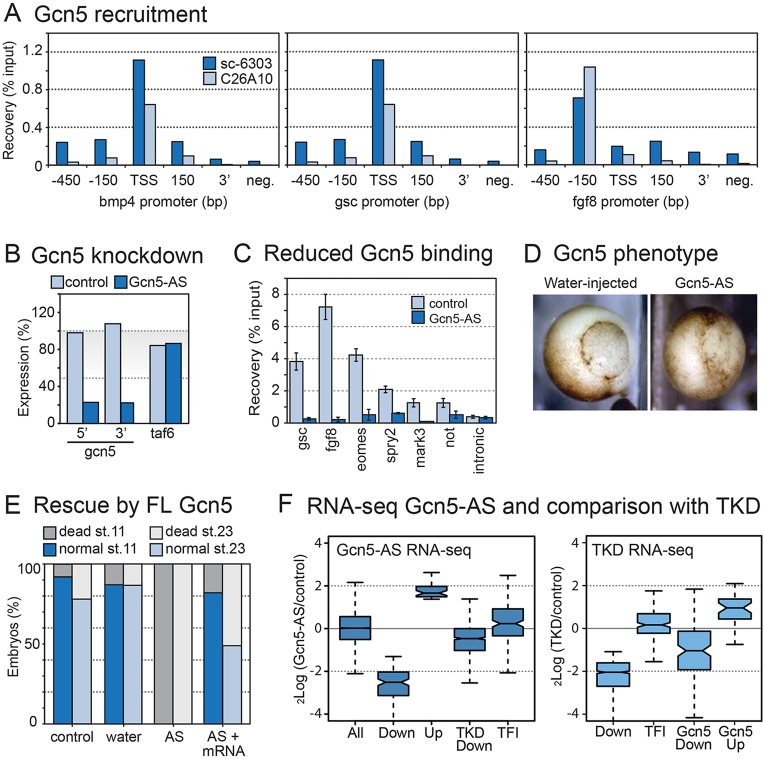
**Analysis of Gcn5 function in early embryos.** A number of TFI genes recruit Gcn5 to their promoter and require Gcn5 for normal expression. (A) Gcn5 binding at different regions around the transcription start site (TSS) of *bmp4*, *gsc* and *fgf8* genes in *X. tropicalis*. ChIP was performed using two different polyclonal anti-GCN5 antibodies. Negative controls (3′ ends of genes and an intergenic region) are indicated. One of the antibodies (C26A10) is highly specific for Gcn5 in western blotting (cf. Fig. S2B). In addition, ChIP signals are reduced in Gcn5-AS-injected embryos (cf. panel C). (B) Depletion of *gcn5* transcripts was verified by RT-qPCR. Expression levels normalized by maternal *gapdh* levels were determined for both 5′ and 3′ regions of the *gcn5* mRNA and compared with a control, *taf6*. (C) Binding of Gcn5 protein to *X. laevis* promoters is reduced upon *gcn5* knockdown, as assessed by ChIP-qPCR using anti-GCN5 C26A10 antibody in control (light blue) and Gcn5-knockdown (dark blue) embryos (stage 10.5). (D) Morphology of control (water-injected) and Gcn5-knockdown (KD, Gcn5-AS injected) embryos showing gastrulation defects at stage 10.5-11. (E) Statistics of rescue experiments performed by co-injecting *in vitro*-transcribed full-length human *GCN5* mRNA (FL Gcn5) together with Gcn5-AS oligos to restore normal development (cf. Fig. S2). Statistics of three independent experiments are summarized. (F) Box plots showing fold change (log_2_) of transcript levels in duplicate samples of Gcn5-KD and control embryos (left panel), shown for all expressed genes, genes with decreased (Down) and increased (Up) transcripts (DEseq FDR 0.1), genes with decreased transcripts in TKD embryos and TFI genes. Right panel shows reciprocal analysis for TKD conditions. The fold changes are depicted in subsets of genes, decreased transcripts in TKD embryos (Down), TFI genes, transcripts that are decreased (Gcn5 Down) or increased (Gcn5 Up) in Gcn5-AS embryos.

The Gcn5-containing SAGA co-activator complex could potentially play a role in transcription of TFI genes, as it is structurally similar to TFIID, is capable of initiating transcription *in vitro* and is recruited to promoter histone modifications (Han et al., 2014; Vermeulen et al., 2010; Wieczorek et al., 1998). Gcn5 was not identified as TBP family insensitive because its mRNA is partly maternally derived; however, *gcn5* mRNA levels are not affected by knockdown of TBP family members (Fig. S2A). To examine the role of Gcn5 in TFI gene expression we investigated Gcn5 binding by ChIP using two different Gcn5-specific antibodies (Fig. 4A). We found Gcn5 binding close to the annotated transcription start site of *bmp4*, *gsc* and *fgf8*, whereas distal regions were negative (Fig. 4A). To explore a potential role of *gcn5* in the transcription of TFI genes, we studied the effect of depletion of *gcn5* mRNA by antisense knockdown using DMED-modified oligonucleotides (Fig. 4B). Concordant with specific antibody recognition of Gcn5 on western blot (Fig. S2B), Gcn5 protein was depleted from promoters in antisense-injected embryos compared with controls using ChIP analysis (Fig. 4C).

In *gcn5* knockdown embryos, an initial blastopore groove was observed on the dorsal side at beginning of gastrulation (stage 10-10.5) but involution was affected and the embryos entered developmental arrest (Fig. 4D) with signs of apoptosis, mostly at the ventral side (Fig. S2C). To assess the specificity of Gcn5 knockdown (KD), we carried out rescue experiments using *in vitro*-transcribed mRNA encoding the human protein. The results showed that the phenotype of antisense-injected embryos can be attributed to knockdown of *gcn5* (Fig. 4E and Fig. S2D-E). Moreover, the phenotype could also be rescued using Gcn5 with a mutated HAT domain, suggesting that HAT activity is not required for early development (Fig. S2D,E).

To determine how embryonic transcription is changed by the knockdown of Gcn5, we generated transcriptome profiles of early gastrula (stage 10.5) control and Gcn5-KD embryos in duplicate by RNA sequencing (Table S4). Among decreased transcripts [125 at false discovery rate (FDR) 0.1] are those associated with gene ontologies of chromatin modifications (e.g. *kdm5b*, *dnmt1* and *ehmt2*), regulation of actin filament polymerization, ubiquitin-dependent degradation and glycogen metabolism (Fig. S2F, Table S5). Among increased transcripts (40 at FDR 0.1) only regulation of transcription was a significant gene ontology (Table S6), notably contributed to by the TFI transcription factor genes *gsc* and *not*. In addition, three other TFI genes were upregulated in Gcn5-KD embryos, representing a small but highly significant overlap (hypergeometric *P*-value 2.8×10^−5^). Moreover, transcripts affected by Gcn5-KD and TKD showed similar responses under these conditions (Fig. 4F); TFI gene transcripts were mildly increased in Gcn5-KD embryos (Wilcoxon signed rank *P*-value 4.4×10^−5^) and transcripts upregulated by Gcn5-KD also increased in TKD embryos (Wilcoxon signed rank *P*-value 6.8×10^−9^). These data demonstrate that Gcn5 is important for early embryonic gene transcription, but do not support a model of obligatory and non-redundant TFI transcription initiation by Gcn5.

### Increased binding of Gcn5 to TFI genes under TKD conditions

Gcn5 has been implicated in targeting activator proteins to the promoters of inducible genes (Cosma et al., 1999), hence we asked whether Gcn5, upon loss of TBP, TLF and TBP2, might contribute to transcription as a co-activator or by supporting basal transcription. Basal transcription is driven by core promoter elements which recruit initiation factors (Juven-Gershon and Kadonaga, 2010). To examine activated transcription in the absence of TBP family members *in vivo*, we used the VP16 transcription activation assay originally developed in yeast (Larschan and Winston, 2001). Although this assay system is artificial, it allowed us to address the mechanistic question of whether a strong activator is required to bypass a requirement for TBP family initiation factors (Fig. S3A). In control embryos, Gal4-VP16 activated transcription vigorously (>10-fold, Fig. S3B). Strikingly, in TKD embryos, VP16 activation also occurred, albeit with some delay. When basal transcription was analyzed (with no Gal4 protein expressed), reporter expression levels appeared to be significantly higher in TKD embryos than in controls, similar to some of the TFI gene transcripts (Fig. 2C). This result indicates that TKD conditions can facilitate both basal transcription driven by the core promoter (Fig. S3B, right panel) and transcription driven by a strong activator.

**Fig. 5.**
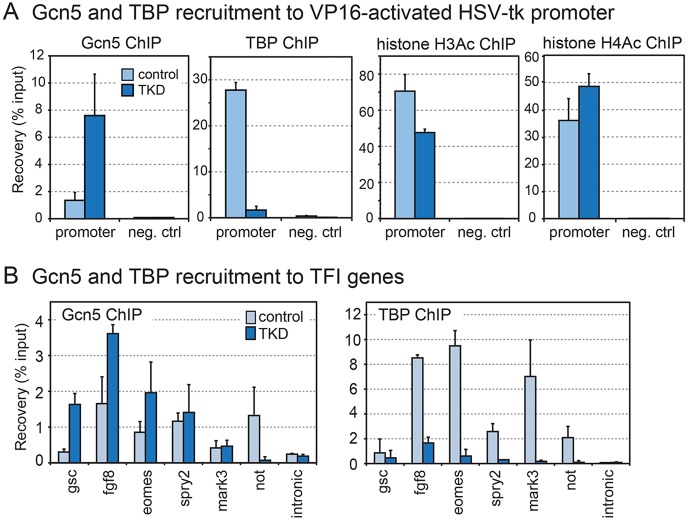
**Increased promoter binding of Gcn5 in TBP family triple-knockdown embryos.** (A) ChIP analysis of Gcn5, TBP and histone H3 and H4 acetylation under VP16 activating conditions in TKD and control embryos. These experiments were performed in a VP16 transcription activation assay combined with TBP family loss-of-function experiments (cf. Fig. S3). Loss of TBP on the promoter in TKD embryos is compensated by an increase in Gcn5 (first two panels). Histones H3 and H4 are acetylated in both TKD and control embryos (second two panels). (B) Gcn5 is recruited to TFI gene promoters in TBP family triple-knockdown embryos. ChIP reveals enhanced Gcn5 binding when TBP and TBP-related factors are depleted in TKD embryos (blue) compared with water-injected controls (light blue). The intronic region of *nadh* gene shows background levels. The *mark3* gene requires normal TBP levels for expression.

To relate transcription levels to promoter binding, we carried out ChIP-qPCR experiments to detect TBP and Gcn5 at the promoter under VP16-activated conditions. Additionally, we quantified acetylated histone H3 and histone H4 in control and TKD embryos. The promoter was bound by TBP and relatively little Gcn5 in control embryos. TBP binding was abolished in TKD embryos (Fig. 5A), as expected while the promoter is still driving robust transcription (Fig. S3B). Strikingly, Gcn5 showed the opposite pattern with more than 7-fold stronger binding to the promoter in TKD embryos compared with control embryos. Moreover, robust histone acetylation was detected in both samples. These results show that both basal and activator-dependent transcription can occur under TKD conditions and that compensatory recruitment of Gcn5 might occur in the absence of TBP and TBP-related factors.

We asked whether a similar compensatory binding is observed at endogenous TFI promoters under TKD conditions. We analyzed Gcn5 binding in TKD embryos and compared it with controls. We confirmed the expected loss of TBP binding in TKD embryos (Fig. 5B). The results show that Gcn5 recruitment to the TFI gene promoters of *gsc*, *fgf8* and *eomes* is significantly higher in TKD embryos than in water-injected controls. Other TFI genes recruit Gcn5, but binding was either unaffected by TKD or decreased. The TBP-dependent *mark3* gene recruited some Gcn5 but this was unchanged upon TBP family depletion, similar to *spry2*. TBP binding was strongly reduced in TKD embryos.

### TFI genes are bound by TFI transcription factors

**Fig. 6.**
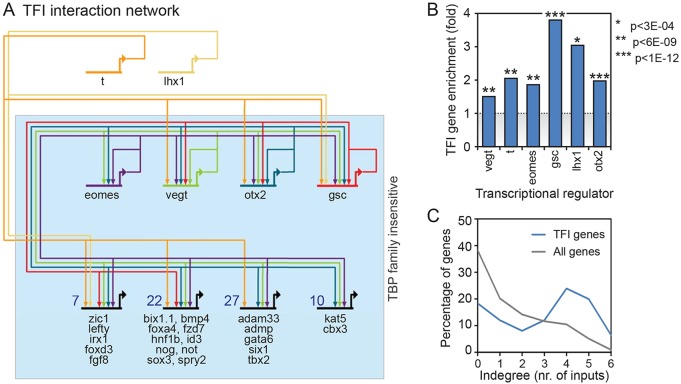
**TFI network analysis.** (A) TFI genomic interaction network (blue box) based on ChIP data of Otx2, Vegt, Eomes, Gsc, T and Lhx1 in *X. tropicalis*. The most common binding combinations with representative TFI genes are depicted on the bottom row. The total number of TFI genes with the same binding combinations is indicated. For a complete overview, see Fig. S4. (B) Over-representation of TFI genes among genes bound by Otx2, Vegt, Eomes, Gsc, T and Lhx1. Hypergeometric *P*-values are indicated. (C) Indegree (number of inputs) of TFI gene transcripts (blue) and all genes (gray). TFI gene transcripts are often bound by four or more of the transcription factors Otx2, Vegt, Eomes, Gsc, T and Lhx1.

To further assess the contribution of transcriptional regulators to TFI transcription, we examined the involvement of TFI genes in the gene regulatory network of mesendoderm specification (Koide et al., 2005). Strikingly, the mesendoderm specification network is enriched for TFI genes (8 out of 23 mesendoderm specification network genes, hypergeometric *P*-value 9.3×10^−13^, Fig. S4A). It has been reported that the T-box binding factors T (Xbra), Vegt and Eomes, which are expressed in the marginal zone, are co-recruited at the regulatory regions of target genes (Gentsch et al., 2013). Also the recruitment of the organizer-expressed homeobox factors Gsc, Otx2 and Lhx1 (Lim1) has been studied in *X. tropicalis* embryos by ChIP sequencing (Yasuoka et al., 2014). Four of these factors (Vegt, Eomes, Otx2, Gsc) do not require the TBP family for efficient transcription initiation; therefore, these factors could play a role in TFI transcription. Thus, we mapped their binding sites to genes to construct a genomic interaction network (see Materials and Methods). Strikingly, 103 of 126 (82%) of named TFI genes had one or more of the six transcription factors (T, Eomes, Vegt, Gsc, Otx2, Lhx1) bound to their locus, and the same 82% were bound by one or more of the four TFI transcription factors (Eomes, Vegt, Gsc, Otx2; Fig. S4B). Furthermore, Vegt, Otx2 and Gsc form a highly interconnected network that displays all possible mutual interactions between these factors and the genes encoding them, with intertwined putative auto-regulatory, feed-forward and multi-component circuitry (Fig. 6A). TFI genes are more frequently bound by these transcription factors than the set of all genes (Fig. 6B, hypergeometric *P*-values between 3×10^−4^ and 1×10^−16^). Downstream TFI genes show a higher degree of coordinated binding; many are bound by four, five or six transcription factors (Fig. 6A,C). We compared the effects of Gsc knockdown and Lhx1/Otx2/Otx5 triple knockdown (Yasuoka et al., 2014) on TFI gene expression (Fig. S4B). TFI genes tend to be upregulated in *gsc* morphant embryos (65 genes; Wilcoxon *P*-value, 1×10^−4^). The effects of combined Lhx1, Otx2 and Otx5 knockdown (only *otx2* is a TFI gene) are less clear. Whereas organizer-expressed TFI genes *gsc*, *foxa4* and *admp* are expressed at lower levels in the triple morphant (*lhx1*/*otx2*/*otx5*) embryos, *foxd3*, *not*, *rhob* and *otx2* itself are upregulated (Fig. S4B). Additional interactions, possibly including those mediated by other TFI transcription factors, might explain these non-parsimonious gene regulatory relationships. Together, the data uncover a TFI gene network centered on putative auto-regulatory and multicomponent circuitry of T-box, Otx2 and Gsc binding; the majority of the TFI genes bind these TFI transcriptional regulators.

## DISCUSSION

TBP is a key transcription initiation factor in eukaryotes; however, genomes of multicellular organisms generally encode one or more TBP-related factors (TLF and TBP2 in vertebrates), as well as additional TFIIA-like and TAF-like factors (Akhtar and Veenstra, 2011; Duttke et al., 2014; Goodrich and Tjian, 2010; Müller and Tora, 2004, 2014). Indeed, although TBP is essential for viability, not all transcription requires TBP in fish, frog and mouse embryos (Martianov et al., 2002b; Müller et al., 2001; Veenstra et al., 2000). In yeast, and on human promoters *in vitro*, TBP binding is a rate-limiting step for transcription (Kim and Iyer, 2004; Klein and Struhl, 1994; Wu and Chiang, 2001). There are known differences in core promoter recognition within the TBP family; TLF/TRF2 recognizes TCT, Inr and DPE core promoter elements (Kedmi et al., 2014; Wang et al., 2014), whereas TBP2 recognizes TATA boxes in a similar manner to TBP (Bártfai et al., 2004; Jallow et al., 2004). Apart from these differences, TBP-related factors are thought to support transcription initiation in a comparable fashion. Concordantly, when *tbp*, *tlf* and *tbp2* mRNA transcripts are ablated in *Xenopus* embryos, more than 90% of actively transcribed genes are affected (Fig. 1D). However, we have identified and characterized a small cluster of transcripts that are not affected by the levels of the three TBP family members. They are referred to as TBP family-insensitive (TFI) because transcript levels do not (or only marginally) change upon ablation of TBP family members. TFI genes are actively transcribed and this transcription cannot be explained by redundancy between the TBP family members. They efficiently recruit the initiating form of RNAPII to their promoters, indicating that TFI genes initiate transcription through a non-canonical mechanism (Fig. 2). Even though TFI genes do not require Gcn5, recruitment of Gcn5 to the promoter increases with the loss of TBP binding upon knockdown of all three TBP family members (TKD, Fig. 5). This substitution of TBP by Gcn5 might reflect a high degree of functional plasticity in transcription initiation. Experiments using the strong VP16 activator indicated that this may not simply involve a bypass of the basal transcription machinery by strong activators (which could potentially recruit RNAPII directly), but can occur without any activators (Fig. S3). We have not determined whether Gcn5 binds as a complex to TFI promoters, but the results suggest that the SAGA complex, which is structurally similar to TFIID (Han et al., 2014), could play a role in this process. Like TFIID, SAGA binds to the H3K4me3 histone modification that is found at promoters (Vermeulen et al., 2010). In yeast, TFIID and SAGA are partially redundant, but some genes require either TFIID or SAGA subunits for normal expression (Huisinga and Pugh, 2004; Lee et al., 2000; Li et al., 2000; Timmers and Tora, 2005). Our Gcn5 knockdown analysis did not reveal a non-redundant requirement for Gcn5 in TFI gene transcription. In the absence of TBP family members, the activation of TFI genes is preserved and switches to a TBP-free regulation. Some promoters opportunistically recruit Gcn5 under these conditions, suggesting that Gcn5/SAGA and TBP/TFIID compete with each other, potentially through interactions of activators with shared TAF subunits.

The TFI genes identified in this study are enriched for mesoderm and organizer expression and function (Fig. 3). Although this might partly represent a bias resulting from the developmental stage in which the analysis was performed, it should be noted that TFI genes form a relatively small and highly selective subset of transcripts that show similar characteristics in terms of developmental activation and active transcription at this stage. Strikingly, a large majority (82%) of TFI genes are bound by one or more of the TFI transcription factors Vegt, Eomes, Otx2 and Gsc (Fig. 6, Fig. S4). It should be noted that these TFI transcription factor genes are expressed in spatially overlapping domains, with the T-box genes mainly expressed in the marginal zone (and vegetal pole, *vegt*), and *otx2* and *gsc* expressed in the organizer. In this genomic interaction network, no attempt has been made to infer activating or repressive influences. T-box factors tend to activate mesodermal target genes (Gentsch et al., 2013). Otx2 and Lhx1 targets are activated within the organizer region, whereas Gsc is a repressor and co-recruitment of Gsc and Otx2 might also lead to repression (Artinger et al., 1997; Danilov et al., 1998; Latinkic et al., 1997; Mochizuki et al., 2000; Yamamoto et al., 2003; Yasuoka et al., 2014). The four TFI transcription factors Eomes, Vegt, Otx2 and Gsc form a densely connected core network (Fig. 6A); two combinations of three factors (Eomes-Vegt-Otx2 and Vegt-Otx2-Gsc) constitute fully connected triads with all possible mutual interactions including auto-regulatory interactions. Mutual positive interactions (positive feedback) confer switch behavior, whereas mutual negative-feedback interactions function as fast-response circuitry but also can contribute to switch behavior and formation of expression boundaries (Alon, 2007; Davidson, 2010; Shoval and Alon, 2010). There are additional feed-forward loop and multi-component loop modules embedded in the network, and its high degree of connectivity has attractor properties that are expected for circuitry that confers cellular identity. The positively acting Eomes-Vegt and Eomes-Vegt-Otx2 modules probably lock in mesodermal and organizer identity in the marginal zone and organizer regions, respectively. This highly connected mesoderm and organizer subnetwork not only has interesting network properties, its mechanisms of transcription initiation are also highly flexible and do not strictly require TBP, TLF or TBP2. The TBP family-independent T-box-Otx2-Gsc core network of transcription factors identified here might therefore provide autonomous regulatory control to TFI genes, facilitated by the plasticity in transcription initiation. The binding properties of Gcn5, as well as the finding that the core promoter might be sufficient to drive TFI gene transcription, suggests that this is not merely a byproduct of strong activation but an inherent property of the transcription machinery. The plasticity of transcription initiation could provide robustness to the developmental network and accommodate the highly diverse gene regulatory requirements of the developmental program.

## MATERIALS AND METHODS

### Animals

Outbred *Xenopus laevis* (age 1-5 years) were used for *in vitro* fertilization. All procedures were performed after obtaining approval from the Radboud University Ethics Committee for Experimental Animal Research (Ru-dec 2014-122).

### Antisense knockdown and Gcn5 rescue

Knockdown of TBP, TLF and TBP2 was performed as reported previously (Jacobi et al., 2007; Jallow et al., 2004; Veenstra et al., 2000). Stable antisense (AS) DNA oligonucleotides were synthesized with dimethyl-ethylene-diamine (DMED) modifications (Dagle and Weeks, 2001). For TKD of TBP family members, 0.8 ng TBP-AS, 1.2 ng TBP2-AS and 1.0 ng TLF-AS was co-injected into 1-cell stage embryos. For knockdown of *gcn5* (*kat2a*) mRNA, a DMED-modified oligonucleotide Gcn5-AS[BJ220], antisense to position 220-237 of the BJ072879 EST, was designed: 5′-T+C+A+A+A+CACAAGC+C+G+G+G+T+T-3′ (+ denotes DMED-modified linkages). 0.8 ng of Gcn5-AS[BJ220] was used for knockdown. In rescue experiments, mRNA transcribed from pcDNA3.1-Myc-His-hGCN5 was used (Yanagisawa et al., 2002). To express catalytically dead ΔHAT Gcn5, pcDNA3.1-Flag-hGCN5-ΔHAT construct was used with E575A and D615A point mutations (Orpinell et al., 2010). To inhibit transcription, 50 ng α-amanitin per embryo was injected. *In situ* TUNEL (Tdt-mediated dUTP nick end labeling) analysis for detection of apoptosis was performed in whole mount as described (Veenstra et al., 1998).

### Gene expression analysis

RNA was isolated using Trizol (Life Technologies) and RNeasy columns (Qiagene). Reverse transcription was performed with Superscript II (Life Technologies) and cDNAs were measured using iQ SYBR Green Supermix and a C1000 thermal cycler CFX96 reader system (Bio-Rad). For microarray analysis, 10 µg total RNA was processed with Affymetrix one-cycle kit. Labeling and hybridization was done according to Affymetrix GeneChip instructions. Microarray data were scaled with GeneChip Operating Software (GCOS) by comparing samples with stage 10.5 control embryos and transferred to Spotfire Decision Site 7.3 for analysis. K-means clustering (*k*=8, Pearson correlation) was performed using TM4 MeV (http://www.tm4.org/) (Saeed et al., 2003).

For RNA sequencing, total RNA was isolated and depleted of ribosomal RNA, as previously described (Paranjpe et al., 2013). Libraries were prepared with the Kapa Hyper Prep kit (Kapa Biosystems). Reads were mapped to the reference *X. laevis* genome assembly JGI9.1, downloaded from Xenbase (James-Zorn et al., 2015), using STAR (Dobin et al., 2013) and allowing one mismatch. Differential transcripts were identified using DEseq (Anders and Huber, 2010), controlling the false discovery rate at 10% using the adjusted *P*-value.

For analysis of localized expression of TFI gene transcripts, GEO GSE8990 data (Tanegashima et al., 2009) was downloaded and normalized in R/Bioconductor using RMA limma (Irizarry et al., 2003). Animal-vegetal and dorsal-ventral log_2_ ratios were calculated and differentially expressed transcripts were filtered based on log_2_ ratio differences greater than 1.5. A comparison of TFI gene transcripts with *nog*-*dkk1*-induced transcripts was performed based on Affymetrix IDs provided in Hufton et al. (2006). For gene ontology (GO) term analysis Affymetrix IDs were mapped to Xenbase gene names and enrichment analysis was carried out using DAVID (Huang et al., 2007). GO terms were filtered using number of term items ≥10, FDR ≤0.05 and fold enrichment ≥4.

### Gene network analysis

Regulatory interactions of the mesendoderm specification network (Koide et al., 2005) were visualized using BioTapestry (Longabaugh, 2012). For analysis of T, Vegt, Eomes, Gsc, Lhx1 and Otx2 binding, ChIP-seq data (Gentsch et al., 2013; Yasuoka et al., 2014) was mapped to *X. tropicalis* assembly 7.1. Peaks were called using MACS2 (Zhang et al., 2008) with q0.001. To obtain high-confidence peak sets, RPKM read coverage values were calculated and peaks were filtered for RPKM greater than 99% of the RPKM in the input tracks for the same peak regions. Filtered peaks were assigned to GREAT regions (McLean et al., 2010) of xtev 3.4 genes (Paranjpe et al., 2013). The *P*-value of the hypergeometric distribution was used to determine statistical significance of over-representation.

### Chromatin immunoprecipitation

Chromatin immunoprecipitation (ChIP) was performed as described previously (Akkers et al., 2012; Jallow et al., 2004). Antibodies were bound to 1/10 volume of magnetic beads in suspension and manufacturer's instructions were followed (Dynabeads Magnetic Separation Technology). The following antibodies were used: goat polyclonal anti-GCN5 (N-18) (sc-6303, Santa-Cruz Biotechnology; 1:100; 2 μg/ChIP) and rabbit monoclonal C26A10 anti-GCN5 (3305S, Cell Signaling; 10× concentrated in 1×PBS without preservatives, 1:100 diluted for western blot; and 1:40, 1-2 μg/ChIP), anti-RNAPII-CTD-phospho-Ser5 3E8 (ActiveMotif; 1:40; 5 μg/ChIP), anti-RNAPII-CTD 8WG16 (MMS-126R, Covance/BioLegend; 1:100, 1 μg/ChIP), anti-acetyl-histone H4 (06-866, Millipore; 1:100, 1 μg/ChIP), anti-acetyl histone H3 (06-599, Millipore; 1:100; 1 μg/ChIP) and mouse monoclonal SL30 anti-TBP (SL-30-3-563, EMD Millipore; 1:40; 10 μg/ChIP) (Jallow et al., 2004; Ruppert et al., 1996). De-crosslinking and elution of DNA was performed in one step using iPure kit (Diagenode). Real-time quantitative PCR was carried using iQ SYBR Green Supermix and C1000 thermal cycler CFX96 reader system (Bio-Rad).

### VP16 transcription activation assay in embryos

Embryos at 1-cell stage were injected with 0.3 ng *in vitro*-transcribed mRNAs (MessageMachine Kit, Ambion) to express a fusion protein of the Gal4 DNA-binding domain and the VP16 activation domain (Gal4-VP16) or Gal4 DNA-binding domain (Gal 4DBD) to obtain activating and non-activating conditions on 0.15 ng of a co-injected DNA template containing an HSV-tk promoter with five Gal4 binding sites. CAT reporter transcripts were measured using RT-qPCR.

## References

[DEV127936C1] Adelman K. and Lis J. T. (2012). Promoter-proximal pausing of RNA polymerase II: emerging roles in metazoans. *Nat. Rev. Genet.* 13, 720-731. 10.1038/nrg3293 22986266 PMC3552498

[DEV127936C2] Akhtar W. and Veenstra G. J. C. (2009). TBP2 is a substitute for TBP in Xenopus oocyte transcription. *BMC Biol.* 7, 45 10.1186/1741-7007-7-45 19650908 PMC2731028

[DEV127936C3] Akhtar W. and Veenstra G. J. C. (2011). TBP-related factors: a paradigm of diversity in transcription initiation. *Cell Biosci.* 1, 23 10.1186/2045-3701-1-23 21711503 PMC3142196

[DEV127936C4] Akkers R. C., Jacobi U. G. and Veenstra G. J. C. (2012). Chromatin immunoprecipitation analysis of Xenopus embryos. *Methods Mol. Biol.* 917, 279-292. 10.1007/978-1-61779-992-1_17 22956095

[DEV127936C5] Alon U. (2007). Network motifs: theory and experimental approaches. *Nat. Rev. Genet.* 8, 450-461. 10.1038/nrg2102 17510665

[DEV127936C6] Anders S. and Huber W. (2010). Differential expression analysis for sequence count data. *Genome Biol.* 11, R106 10.1186/gb-2010-11-10-r106 20979621 PMC3218662

[DEV127936C7] Artinger M., Blitz I., Inoue K., Tran U. and Cho K. W. Y. (1997). Interaction of goosecoid and brachyury in Xenopus mesoderm patterning. *Mech. Dev.* 65, 187-196. 10.1016/S0925-4773(97)00073-7 9256355

[DEV127936C8] Bártfai R., Balduf C., Hilton T., Rathmann Y., Hadzhiev Y., Tora L., Orbán L. and Müller F. (2004). TBP2, a vertebrate-specific member of the TBP family, is required in embryonic development of zebrafish. *Curr. Biol.* 14, 593-598. 10.1016/j.cub.2004.03.034 15062100

[DEV127936C9] Bonnet J., Wang C.-Y., Baptista T., Vincent S. D., Hsiao W.-C., Stierle M., Kao C.-F., Tora L. and Devys D. (2014). The SAGA coactivator complex acts on the whole transcribed genome and is required for RNA polymerase II transcription. *Genes Dev.* 28, 1999-2012. 10.1101/gad.250225.114 25228644 PMC4173158

[DEV127936C10] Cosma M. P., Tanaka T. and Nasmyth K. (1999). Ordered recruitment of transcription and chromatin remodeling factors to a cell cycle- and developmentally regulated promoter. *Cell* 97, 299-311. 10.1016/S0092-8674(00)80740-0 10319811

[DEV127936C11] Crowley T. E., Hoey T., Liu J.-K., Jan Y. N., Jan L. Y. and Tjian R. (1993). A new factor related to TATA-binding protein has highly restricted expression patterns in Drosophila. *Nature* 361, 557-561. 10.1038/361557a0 8429912

[DEV127936C12] Dagle J. M. and Weeks D. L. (2001). Oligonucleotide-based strategies to reduce gene expression. *Differentiation* 69, 75-82. 10.1046/j.1432-0436.2001.690201.x 11798068

[DEV127936C13] Danilov V., Blum M., Schweickert A., Campione M. and Steinbeisser H. (1998). Negative autoregulation of the organizer-specific homeobox gene goosecoid. *J. Biol. Chem.* 273, 627-635. 10.1074/jbc.273.1.627 9417125

[DEV127936C14] Dantonel J.-C., Quintin S., Lakatos L., Labouesse M. and Tora L. (2000). TBP-like factor is required for embryonic RNA polymerase II transcription in C. elegans. *Mol. Cell* 6, 715-722. 10.1016/S1097-2765(00)00069-1 11030350

[DEV127936C15] Davidson E. H. (2010). Emerging properties of animal gene regulatory networks. *Nature* 468, 911-920. 10.1038/nature09645 21164479 PMC3967874

[DEV127936C16] Dobin A., Davis C. A., Schlesinger F., Drenkow J., Zaleski C., Jha S., Batut P., Chaisson M. and Gingeras T. R. (2013). STAR: ultrafast universal RNA-seq aligner. *Bioinformatics* 29, 15-21. 10.1093/bioinformatics/bts635 23104886 PMC3530905

[DEV127936C17] Duttke S. H. C., Doolittle R. F., Wang Y.-L. and Kadonaga J. T. (2014). TRF2 and the evolution of the bilateria. *Genes Dev.* 28, 2071-2076. 10.1101/gad.250563.114 25274724 PMC4180970

[DEV127936C18] Fukuda M., Takahashi S., Haramoto Y., Onuma Y., Kim Y.-J., Yeo C.-Y., Ishiura S. and Asashima M. (2010). Zygotic VegT is required for Xenopus paraxial mesoderm formation and is regulated by Nodal signaling and Eomesodermin. *Int. J. Dev. Biol.* 54, 81-92. 10.1387/ijdb.082837mf 20013651

[DEV127936C111] Gazdag E., Rajkovic A., Torres-Padilla M. E. and Tora L. (2007). Analysis of TATA-binding protein 2 (TBP2) and TBP expression suggests different roles for the two proteins in regulation of gene expression during oogenesis and early mouse development. *Reproduction* 134, 51-62. 10.1530/REP-06-033 17641088

[DEV127936C19] Gazdag E., Santenard A., Ziegler-Birling C., Altobelli G., Poch O., Tora L. and Torres-Padilla M.-E. (2009). TBP2 is essential for germ cell development by regulating transcription and chromatin condensation in the oocyte. *Genes Dev.* 23, 2210-2223. 10.1101/gad.535209 19759265 PMC2751983

[DEV127936C20] Gentsch G. E., Owens N. D. L., Martin S. R., Piccinelli P., Faial T., Trotter M. W. B., Gilchrist M. J. and Smith J. C. (2013). In vivo T-box transcription factor profiling reveals joint regulation of embryonic neuromesodermal bipotency. *Cell Rep.* 4, 1185-1196. 10.1016/j.celrep.2013.08.012 24055059 PMC3791401

[DEV127936C21] Goodrich J. A. and Tjian R. (2010). Unexpected roles for core promoter recognition factors in cell-type-specific transcription and gene regulation. *Nat. Rev. Genet.* 11, 549-558. 10.1038/nrg2847 20628347 PMC2965628

[DEV127936C22] Han Y., Luo J., Ranish J. and Hahn S. (2014). Architecture of the Saccharomyces cerevisiae SAGA transcription coactivator complex. *EMBO J.* 33, 2534-2546. 10.15252/embj.201488638 25216679 PMC4283410

[DEV127936C23] Hansen S. K., Takada S., Jacobson R. H., Lis J. T. and Tjian R. (1997). Transcription properties of a cell type-specific TATA-binding protein, TRF. *Cell* 91, 71-83. 10.1016/S0092-8674(01)80010-6 9335336

[DEV127936C24] Hart D. O., Raha T., Lawson N. D. and Green M. R. (2007). Initiation of zebrafish haematopoiesis by the TATA-box-binding protein-related factor Trf3. *Nature* 450, 1082-1085. 10.1038/nature06349 18046332 PMC2150749

[DEV127936C25] Huang D. W., Sherman B. T., Tan Q., Collins J. R., Alvord W. G., Roayaei J., Stephens R., Baseler M. W., Lane H. C. and Lempicki R. A. (2007). The DAVID Gene Functional Classification Tool: a novel biological module-centric algorithm to functionally analyze large gene lists. *Genome Biol.* 8, R183 10.1186/gb-2007-8-9-r183 17784955 PMC2375021

[DEV127936C26] Hufton A. L., Vinayagam A., Suhai S. and Baker J. C. (2006). Genomic analysis of Xenopus organizer function. *BMC Dev. Biol.* 6, 27 10.1186/1471-213X-6-27 16756679 PMC1513553

[DEV127936C27] Huisinga K. L. and Pugh B. F. (2004). A genome-wide housekeeping role for TFIID and a highly regulated stress-related role for SAGA in Saccharomyces cerevisiae. *Mol. Cell* 13, 573-585. 10.1016/S1097-2765(04)00087-5 14992726

[DEV127936C28] Irizarry R. A., Hobbs B., Collin F., Beazer-Barclay Y. D., Antonellis K. J., Scherf U. and Speed T. P. (2003). Exploration, normalization, and summaries of high density oligonucleotide array probe level data. *Biostatistics* 4, 249-264. 10.1093/biostatistics/4.2.249 12925520

[DEV127936C29] Jacobi U. G., Akkers R. C., Pierson E. S., Weeks D. L., Dagle J. M. and Veenstra G. J. C. (2007). TBP paralogs accommodate metazoan- and vertebrate-specific developmental gene regulation. *EMBO J.* 26, 3900-3909. 10.1038/sj.emboj.7601822 17703192 PMC1994123

[DEV127936C30] Jallow Z., Jacobi U. G., Weeks D. L., Dawid I. B. and Veenstra G. J. C. (2004). Specialized and redundant roles of TBP and a vertebrate-specific TBP paralog in embryonic gene regulation in Xenopus. *Proc. Natl. Acad. Sci. USA* 101, 13525-13530. 10.1073/pnas.0405536101 15345743 PMC518790

[DEV127936C31] James-Zorn C., Ponferrada V. G., Burns K. A., Fortriede J. D., Lotay V. S., Liu Y., Brad Karpinka J., Karimi K., Zorn A. M. and Vize P. D. (2015). Xenbase: core features, data acquisition, and data processing. *Genesis* 53, 486-497. 10.1002/dvg.22873 26150211 PMC4545734

[DEV127936C32] Juven-Gershon T. and Kadonaga J. T. (2010). Regulation of gene expression via the core promoter and the basal transcriptional machinery. *Dev. Biol.* 339, 225-229. 10.1016/j.ydbio.2009.08.009 19682982 PMC2830304

[DEV127936C33] Kaltenbach L., Horner M. A., Rothman J. H. and Mango S. E. (2000). The TBP-like factor CeTLF is required to activate RNA polymerase II transcription during C. elegans embryogenesis. *Mol. Cell* 6, 705-713. 10.1016/S1097-2765(00)00068-X 11030349

[DEV127936C34] Kedmi A., Zehavi Y., Glick Y., Orenstein Y., Ideses D., Wachtel C., Doniger T., Waldman Ben-Asher H., Muster N., Thompson J. et al. (2014). Drosophila TRF2 is a preferential core promoter regulator. *Genes Dev.* 28, 2163-2174. 10.1101/gad.245670.114 25223897 PMC4180977

[DEV127936C35] Kim J. and Iyer V. R. (2004). Global role of TATA box-binding protein recruitment to promoters in mediating gene expression profiles. *Mol. Cell. Biol.* 24, 8104-8112. 10.1128/MCB.24.18.8104-8112.2004 15340072 PMC515063

[DEV127936C36] Klein C. and Struhl K. (1994). Increased recruitment of TATA-binding protein to the promoter by transcriptional activation domains in vivo. *Science* 266, 280-282. 10.1126/science.7939664 7939664

[DEV127936C37] Koide T., Hayata T. and Cho K. W. Y. (2005). Xenopus as a model system to study transcriptional regulatory networks. *Proc. Natl. Acad. Sci. USA* 102, 4943-4948. 10.1073/pnas.0408125102 15795378 PMC555977

[DEV127936C38] Kopytova D. V., Krasnov A. N., Kopantceva M. R., Nabirochkina E. N., Nikolenko J. V., Maksimenko O., Kurshakova M. M., Lebedeva L. A., Yerokhin M. M., Simonova O. B. et al. (2006). Two isoforms of Drosophila TRF2 are involved in embryonic development, premeiotic chromatin condensation, and proper differentiation of germ cells of both sexes. *Mol. Cell. Biol.* 26, 7492-7505. 10.1128/MCB.00349-06 17015475 PMC1636870

[DEV127936C39] Larschan E. and Winston F. (2001). The S. cerevisiae SAGA complex functions in vivo as a coactivator for transcriptional activation by Gal4. *Genes Dev.* 15, 1946-1956. 10.1101/gad.911501 11485989 PMC312753

[DEV127936C40] Latinkic B. V., Umbhauer M., Neal K. A., Lerchner W., Smith J. C. and Cunliffe V. (1997). The Xenopus Brachyury promoter is activated by FGF and low concentrations of activin and suppressed by high concentrations of activin and by paired-type homeodomain proteins. *Genes Dev.* 11, 3265-3276. 10.1101/gad.11.23.3265 9389657 PMC316753

[DEV127936C41] Lee T. I., Causton H. C., Holstege F. C. P., Shen W.-C., Hannett N., Jennings E. G., Winston F., Green M. R. and Young R. A. (2000). Redundant roles for the TFIID and SAGA complexes in global transcription. *Nature* 405, 701-704. 10.1038/35015104 10864329

[DEV127936C42] Li X.-Y., Bhaumik S. R. and Green M. R. (2000). Distinct classes of yeast promoters revealed by differential TAF recruitment. *Science* 288, 1242-1244. 10.1126/science.288.5469.1242 10817999

[DEV127936C43] Longabaugh W. J. R. (2012). BioTapestry: a tool to visualize the dynamic properties of gene regulatory networks. *Methods Mol. Biol.* 786, 359-394. 10.1007/978-1-61779-292-2_21 21938637

[DEV127936C44] Martianov I., Brancorsini S., Gansmuller A., Parvinen M., Davidson I. and Sassone-Corsi P. (2002a). Distinct functions of TBP and TLF/TRF2 during spermatogenesis: requirement of TLF for heterochromatic chromocenter formation in haploid round spermatids. *Development* 129, 945-955. 11861477 10.1242/dev.129.4.945

[DEV127936C45] Martianov I., Viville S. and Davidson I. (2002b). RNA polymerase II transcription in murine cells lacking the TATA binding protein. *Science* 298, 1036-1039. 10.1126/science.1076327 12411709

[DEV127936C46] McLean C. Y., Bristor D., Hiller M., Clarke S. L., Schaar B. T., Lowe C. B., Wenger A. M. and Bejerano G. (2010). GREAT improves functional interpretation of cis-regulatory regions. *Nat. Biotechnol.* 28, 495-501. 10.1038/nbt.1630 20436461 PMC4840234

[DEV127936C47] Mochizuki T., Karavanov A. A., Curtiss P. E., Ault K. T., Sugimoto N., Watabe T., Shiokawa K., Jamrich M., Cho K. W. Y., Dawid I. B. et al. (2000). Xlim-1 and LIM domain binding protein 1 cooperate with various transcription factors in the regulation of the goosecoid promoter. *Dev. Biol.* 224, 470-485. 10.1006/dbio.2000.9778 10926781

[DEV127936C48] Müller F. and Tora L. (2004). The multicoloured world of promoter recognition complexes. *EMBO J.* 23, 2-8. 10.1038/sj.emboj.7600027 14685269 PMC1271665

[DEV127936C49] Müller F. and Tora L. (2014). Chromatin and DNA sequences in defining promoters for transcription initiation. *Biochim. Biophys. Acta* 1839, 118-128. 10.1016/j.bbagrm.2013.11.003 24275614

[DEV127936C50] Müller F., Lakatos L., Dantonel J.-C., Strähle U. and Tora L. (2001). TBP is not universally required for zygotic RNA polymerase II transcription in zebrafish. *Curr. Biol.* 11, 282-287. 10.1016/S0960-9822(01)00076-8 11250159

[DEV127936C51] Müller F., Zaucker A. and Tora L. (2010). Developmental regulation of transcription initiation: more than just changing the actors. *Curr. Opin. Genet. Dev.* 20, 533-540. 10.1016/j.gde.2010.06.004 20598874

[DEV127936C52] Nagy Z., Riss A., Fujiyama S., Krebs A., Orpinell M., Jansen P., Cohen A., Stunnenberg H. G., Kato S. and Tora L. (2010). The metazoan ATAC and SAGA coactivator HAT complexes regulate different sets of inducible target genes. *Cell. Mol. Life Sci.* 67, 611-628. 10.1007/s00018-009-0199-8 19936620 PMC11115597

[DEV127936C53] Newport J. and Kirschner M. (1982). A major developmental transition in early Xenopus embryos: I. characterization and timing of cellular changes at the midblastula stage. *Cell* 30, 675-686. 10.1016/0092-8674(82)90272-0 6183003

[DEV127936C54] Orpinell M., Fournier M., Riss A., Nagy Z., Krebs A. R., Frontini M. and Tora L. (2010). The ATAC acetyl transferase complex controls mitotic progression by targeting non-histone substrates. *EMBO J.* 29, 2381-2394. 10.1038/emboj.2010.125 20562830 PMC2910275

[DEV127936C55] Paranjpe S. S., Jacobi U. G., van Heeringen S. J. and Veenstra G. J. C. (2013). A genome-wide survey of maternal and embryonic transcripts during Xenopus tropicalis development. *BMC Genomics* 14, 762 10.1186/1471-2164-14-762 24195446 PMC3907017

[DEV127936C56] Ruppert S. M. L., McCulloch V., Meyer M., Bautista C., Falkowski M., Stunnenberg H. G. and Hernandez N. (1996). Monoclonal antibodies directed against the amino-terminal domain of human TBP cross-react with TBP from other species. *Hybridoma* 15, 55-68. 10.1089/hyb.1996.15.55 9064287

[DEV127936C57] Saeed A. I., Sharov V., White J., Li J., Liang W., Bhagabati N., Braisted J., Klapa M., Currier T., Thiagarajan M. et al. (2003). TM4: a free, open-source system for microarray data management and analysis. *Biotechniques* 34, 374-378. 12613259 10.2144/03342mt01

[DEV127936C58] Shoval O. and Alon U. (2010). SnapShot: network motifs. *Cell* 143, 326-326.e1. 10.1016/j.cell.2010.09.050 20946989

[DEV127936C59] Sible J. C., Anderson J. A., Lewellyn A. L. and Maller J. L. (1997). Zygotic transcription is required to block a maternal program of apoptosis in Xenopus embryos. *Dev. Biol.* 189, 335-346. 10.1006/dbio.1997.8683 9299125

[DEV127936C60] Spedale G., Timmers H. T. M. and Pijnappel W. W. M. P. (2012). ATAC-king the complexity of SAGA during evolution. *Genes Dev.* 26, 527-541. 10.1101/gad.184705.111 22426530 PMC3315114

[DEV127936C61] Tanegashima K., Zhao H., Rebbert M. L. and Dawid I. B. (2009). Coordinated activation of the secretory pathway during notochord formation in the Xenopus embryo. *Development* 136, 3543-3548. 10.1242/dev.036715 19793890 PMC2761104

[DEV127936C62] Timmers H. T. M. and Tora L. (2005). SAGA unveiled. *Trends Biochem. Sci.* 30, 7-10. 10.1016/j.tibs.2004.11.007 15653319

[DEV127936C63] Tupler R., Perini G. and Green M. R. (2001). Expressing the human genome. *Nature* 409, 832-833. 10.1038/35057011 11237001

[DEV127936C64] van Heeringen S. J., Akkers R. C., van Kruijsbergen I., Arif M. A., Hanssen L. L. P., Sharifi N. and Veenstra G. J. C. (2014). Principles of nucleation of H3K27 methylation during embryonic development. *Genome Res.* 24, 401-410. 10.1101/gr.159608.113 24336765 PMC3941105

[DEV127936C65] Veenstra G. J. C., Peterson-Maduro J., Mathu M. T., van der Vliet P. C. and Destrée O. H. J. (1998). Non-cell autonomous induction of apoptosis and loss of posterior structures by activation domain-specific interactions of Oct-1 in the Xenopus embryo. *Cell Death Differ.* 5, 774-784. 10.1038/sj.cdd.4400416 10200537

[DEV127936C66] Veenstra G. J. C., Destrée O. H. J. and Wolffe A. P. (1999). Translation of maternal TATA-binding protein mRNA potentiates basal but not activated transcription in Xenopus embryos at the midblastula transition. *Mol. Cell. Biol.* 19, 7972-7982. 10.1128/MCB.19.12.7972 10567523 PMC84882

[DEV127936C67] Veenstra G. J. C., Weeks D. L. and Wolffe A. P. (2000). Distinct roles for TBP and TBP-like factor in early embryonic gene transcription in Xenopus. *Science* 290, 2312-2315. 10.1126/science.290.5500.2312 11125147

[DEV127936C68] Vermeulen M., Eberl H. C., Matarese F., Marks H., Denissov S., Butter F., Lee K. K., Olsen J. V., Hyman A. A., Stunnenberg H. G. et al. (2010). Quantitative interaction proteomics and genome-wide profiling of epigenetic histone marks and their readers. *Cell* 142, 967-980. 10.1016/j.cell.2010.08.020 20850016

[DEV127936C69] Wang L. and Dent S. Y. R. (2014). Functions of SAGA in development and disease. *Epigenomics* 6, 329-339. 10.2217/epi.14.22 25111486 PMC4159956

[DEV127936C70] Wang Y.-L., Duttke S. H. C., Chen K., Johnston J., Kassavetis G. A., Zeitlinger J. and Kadonaga J. T. (2014). TRF2, but not TBP, mediates the transcription of ribosomal protein genes. *Genes Dev.* 28, 1550-1555. 10.1101/gad.245662.114 24958592 PMC4102762

[DEV127936C71] Wieczorek E., Brand M., Jacq X. and Tora L. (1998). Function of TAF(II)-containing complex without TBP in transcription by RNA polymerase II. *Nature* 393, 187-191. 10.1038/30283 9603525

[DEV127936C72] Wu S.-Y. and Chiang C.-M. (2001). TATA-binding protein-associated factors enhance the recruitment of RNA polymerase II by transcriptional activators. *J. Biol. Chem.* 276, 34235-34243. 10.1074/jbc.M102463200 11457828

[DEV127936C73] Yamamoto S., Hikasa H., Ono H. and Taira M. (2003). Molecular link in the sequential induction of the Spemann organizer: direct activation of the cerberus gene by Xlim-1, Xotx2, Mix.1, and Siamois, immediately downstream from Nodal and Wnt signaling. *Dev. Biol.* 257, 190-204. 10.1016/S0012-1606(03)00034-4 12710967

[DEV127936C74] Yanagisawa J., Kitagawa H., Yanagida M., Wada O., Ogawa S., Nakagomi M., Oishi H., Yamamoto Y., Nagasawa H., McMahon S. B. et al. (2002). Nuclear receptor function requires a TFTC-type histone acetyl transferase complex. *Mol. Cell* 9, 553-562. 10.1016/S1097-2765(02)00478-1 11931763

[DEV127936C75] Yasuoka Y., Suzuki Y., Takahashi S., Someya H., Sudou N., Haramoto Y., Cho K. W., Asashima M., Sugano S. and Taira M. (2014). Occupancy of tissue-specific cis-regulatory modules by Otx2 and TLE/Groucho for embryonic head specification. *Nat. Commun.* 5, 4322 10.1038/ncomms5322 25005894 PMC4805429

[DEV127936C76] Zhang D., Penttila T.-L., Morris P. L., Teichmann M. and Roeder R. G. (2001). Spermiogenesis deficiency in mice lacking the Trf2 gene. *Science* 292, 1153-1155. 10.1126/science.1059188 11352070

[DEV127936C77] Zhang Y., Liu T., Meyer C. A., Eeckhoute J., Johnson D. S., Bernstein B. E., Nussbaum C., Myers R. M., Brown M., Li W. et al. (2008). Model-based analysis of ChIP-Seq (MACS). *Genome Biol.* 9, R137 10.1186/gb-2008-9-9-r137 18798982 PMC2592715

[DEV127936C78] Zhao H., Tanegashima K., Ro H. and Dawid I. B. (2008). Lrig3 regulates neural crest formation in Xenopus by modulating Fgf and Wnt signaling pathways. *Development* 135, 1283-1293. 10.1242/dev.015073 18287203 PMC2749967

